# Correction: Constructing collective identities and solidarity in premiers’ early speeches on COVID-19: a global perspective

**DOI:** 10.1057/s41599-021-00841-7

**Published:** 2021-06-24

**Authors:** Martina Berrocal, Michael Kranert, Paola Attolino, Júlio Antonio Bonatti Santos, Sara Garcia Santamaria, Nancy Henaku, Aimée Danielle Lezou Koffi, Camilla Marziani, Viktorija Mažeikienė, Dasniel Olivera Pérez, Kumaran Rajandran, Aleksandra Salamurović

**Affiliations:** 1 Friedrich Schiller University, Jena, Germany; 2 University of Southhampton, Southhampton, United Kingdom; 3 University of Salerno, Salerno, Italy; 4 Federal University of São Carlos, São Carlos, Brazil; 5 Universitat Jaume Castelló, Castelló, Spain; 6 Michigan Technological University, Michigan, USA; 7 Université Félix Houphouët-Boigny, Côte d’Ivoire, Ivory Coast; 8 University of Bologna, Bologna, Italy; 9 Mykolas Romeris University Vilnius, Vilnius, Lithuania; 10 University of Havana, Havana, Cuba; 11 School of Humanities, Universiti Sains Malaysia, George Town, Malaysia; 12 Friedrich Schiller University, Jena, Germany

**Keywords:** Language and linguistics, Politics and international relations

Correction to: *Humanities and Social Sciences Communications* 10.1057/s41599-021-00805-x, published online 27 May 2021.

The affiliation of one of the authors was wrongly stated as:

Kumaran Rajandran, School of Humanities, Universiti Sains, Sains, Malaysia

This has now been corrected to:

Kumaran Rajandran, School of Humanities, Universiti Sains Malaysia, George Town, Malaysia

This has been corrected in all versions of the paper.

